# Incidence of human brucellosis in the Kilimanjaro Region of Tanzania in the periods 2007–2008 and 2012–2014

**DOI:** 10.1093/trstmh/try033

**Published:** 2018-04-25

**Authors:** Manuela Carugati, Holly M Biggs, Michael J Maze, Robyn A Stoddard, Shama Cash-Goldwasser, Julian T Hertz, Jo E B Halliday, Wilbrod Saganda, Bingileki F Lwezaula, Rudovick R Kazwala, Sarah Cleaveland, Venance P Maro, Matthew P Rubach, John A Crump

**Affiliations:** 1 Division of Infectious Diseases, Duke University Medical Center, Durham, NC, USA; 2 Kilimanjaro Christian Medical Centre, Moshi, Tanzania; 3 Division of Infectious Diseases, San Gerardo Hospital, Monza, Italy; 4 Centre for International Health, University of Otago, Dunedin, New Zealand; 5 Centers for Disease Control and Prevention, Bacterial Special Pathogens Branch, Atlanta, GA, USA; 6 Duke Global Health Institute, Duke University, Durham, NC, USA; 7 Boyd Orr Centre for Population and Ecosystem Health, Institute of Biodiversity, Animal Health and Comparative Medicine, University of Glasgow, Glasgow, UK; 8 Mawenzi Regional Referral Hospital, Moshi, Tanzania; 9 Sokoine University of Agriculture, Morogoro, Tanzania; 10 Kilimanjaro Christian Medical University College, Moshi, Tanzania

**Keywords:** Brucellosis, Incidence, Tanzania

## Abstract

**Background:**

Brucellosis causes substantial morbidity among humans and their livestock. There are few robust estimates of the incidence of brucellosis in sub-Saharan Africa. Using cases identified through sentinel hospital surveillance and health care utilization data, we estimated the incidence of brucellosis in Moshi Urban and Moshi Rural Districts, Kilimanjaro Region, Tanzania, for the periods 2007–2008 and 2012–2014.

**Methods:**

Cases were identified among febrile patients at two sentinel hospitals and were defined as having either a 4-fold increase in *Brucella* microscopic agglutination test titres between acute and convalescent serum or a blood culture positive for *Brucella* spp. Findings from a health care utilization survey were used to estimate multipliers to account for cases not seen at sentinel hospitals.

**Results:**

Of 585 patients enrolled in the period 2007–2008, 13 (2.2%) had brucellosis. Among 1095 patients enrolled in the period 2012–2014, 32 (2.9%) had brucellosis. We estimated an incidence (range based on sensitivity analysis) of brucellosis of 35 (range 32–93) cases per 100 000 persons annually in the period 2007–2008 and 33 (range 30–89) cases per 100 000 persons annually in the period 2012–2014.

**Conclusions:**

We found a moderate incidence of brucellosis in northern Tanzania, suggesting that the disease is endemic and an important human health problem in this area.

## Introduction

Brucellosis is a zoonosis and can cause fever and substantial morbidity among humans and their livestock.^1^ Estimating the burden of human brucellosis is challenging because of underrecognition by health care providers, limited availability of appropriate laboratory diagnostics and difficult access to health care among populations most at risk for brucellosis.^2^ As a consequence, the incidence of brucellosis is uncertain in many sub-Saharan African countries, resulting in few data to guide the allocation of resources for public health interventions and disease control.^3^

Despite the lack of a comprehensive assessment of brucellosis incidence, several studies demonstrate that *Brucella* infection is not uncommon in Tanzania, and more generally in sub-Saharan Africa.^4,5^ Bouley et al.^4^ reported brucellosis among 16 (3.5%) of 870 febrile Tanzanian patients, while Shellings et al.^5^ estimated a brucellosis seroprevalence of 3.8% among nomadic communities in Chad. Infections with *Brucella* are characterized by a low case fatality ratio of approximately 1%, but brucellosis may progress to chronic musculoskeletal, neurologic and cardiovascular complications in humans.^1,6^ Furthermore, since brucellosis is associated with abortion, premature birth and reduced milk production among animals, brucellosis can have a negative economic impact in low-resource settings where livestock is a source of food security and income.^7^ Estimating the incidence of brucellosis in sub-Saharan Africa is a key element for determining the disease burden and the allocation of disease control resources. Utilizing data from two rounds of sentinel site surveillance and health care utilization data, we aimed to estimate the longitudinal incidence of human brucellosis in Moshi Urban and Moshi Rural Districts, Kilimanjaro Region, Tanzania during the periods 2007–2008 and 2012–2014.

## Materials and methods

### Study design

A widely used multiplier study method was applied to estimate the incidence of brucellosis.^8–10^ Our study utilized hospital-based fever surveillance at the two major referral hospitals in the Kilimanjaro Region of Tanzania and a health care utilization survey performed in two districts of the Kilimanjaro Region, the Moshi Urban District and the Moshi Rural District.

### Hospital-based fever surveillance

#### Setting

**Figure 1.**
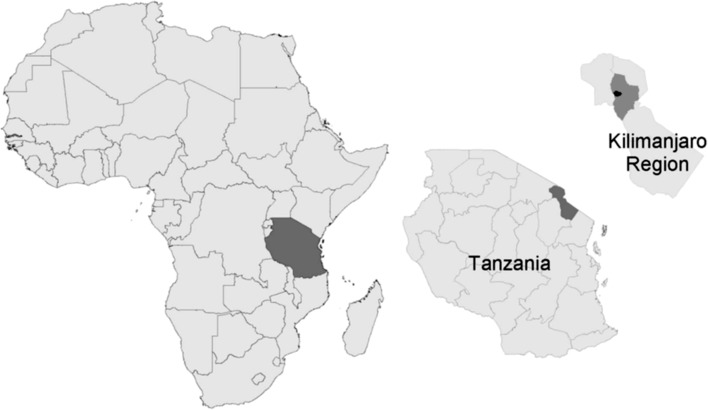
Africa, Tanzania and Kilimanjaro Region. Moshi Rural District shown in dark grey and Moshi Urban District in black in the Kilimanjaro Region inset. Modified from Biggs HM, Hertz JT, Munishi OM, et al. Estimating leptospirosis incidence using hospital-based surveillance and a population-based health care utilization survey in Tanzania. PLoS Negl Trop Dis. 2013;7(12):e2589.

Fever surveillance was conducted at two referral hospitals in Moshi. Moshi is situated in the Kilimanjaro Region of Tanzania at an elevation of approximately 890 m (Figure 1). The climate in Moshi is tropical, with rainy seasons from October through December and March through May. Aside from urban Moshi, the region is rural. The Kilimanjaro Christian Medical Centre (KCMC) is a 450-bed hospital and the zonal referral centre for several regions in northern Tanzania. Mawenzi Regional Referral Hospital (MRRH) is a 300-bed hospital and the referral centre for the Kilimanjaro Region.^11^

#### Study population

Adult and paediatric febrile patients were prospectively enrolled at KCMC and MRRH from 17 September 2007 through 31 August 2008 and from 20 February 2012 through 28 May 2014.^12–14^ During the period 2007–2008 only inpatients were involved in the study: specifically, consecutive febrile adult and paediatric inpatients at KCMC and consecutive febrile adult inpatients at MRRH were included in the study within 24 h of hospital presentation. During the period 2012–2014 both inpatients and outpatients were involved in the study: specifically, consecutive febrile adult inpatients at KCMC, consecutive febrile adult and paediatric inpatients at MRRH and every second febrile adult or paediatric outpatient at MRRH were included in the study within 24 h of hospital presentation. Fever was defined as follows: (1) period 2007–2008, adult inpatients: oral temperature ≥38.0°C; (2) period 2007–2008, paediatric inpatients: history of fever in the past 48 h, an axillary temperature ≥37.5°C or a rectal temperature ≥38.0°C; (3) period 2012–2014, inpatients: a history of fever within the previous 72 h, an axillary temperature >37.5°C or a tympanic, oral or rectal temperature ≥38.0°C; (4) period 2012–2014, outpatients: an axillary temperature >37.5°C or a tympanic, oral or rectal temperature ≥38.0°C.^12–14^

#### Study procedures

Enrolment occurred Monday through Friday. Demographic information, including the participant’s district and village of residence, was collected. BacT/ALERT (bioMerieux, Marcy l’Etoile, France) blood culture bottles were inoculated (Standard Aerobic for adults and Paediatric Fastidious Antibiotic Neutralization for participants <13 years of age) and acute sera were collected. A convalescent serum sample was collected 4–6 weeks after study enrolment. Interhospital transfer was recorded only for the period 2007–2008.

#### Diagnosis of brucellosis

Acute and convalescent serum samples were sent to the US Centers for Disease Control and Prevention (CDC) for serologic analysis for brucellosis by microscopic agglutination test (MAT) using standardized *Brucella abortus* strain 1119-3 killed antigen (National Veterinary Services Laboratory [NVSL], Ames, IA, USA) at a 1:25 working dilution. Samples were inoculated into U-bottom plates and incubated at 26°C. High-positive, low-positive and negative control sera (NVSL) were also included for each test run. Results were read on a Scienceware Plate Reader (Bel-Art Products, Wayne, NJ, USA). Inoculated blood culture bottles were loaded into the BacT/ALERT 3D Microbial Detection System (bioMerieux, Marcy l’Etoile, France), where they were incubated for 5 days.^15,16^ Cases were defined by a 4-fold or greater increase in the *B. abortus* MAT antibody titre between acute and convalescent serum or by isolation of *Brucella* spp. from blood cultures.^17^

### Health care utilization survey

A health care utilization survey was performed in the Moshi Urban District (population 184 292) and Moshi Rural District (population 466 737) of the Kilimanjaro Region between 13 June 2011 and 22 July 2011, as previously described.^18^ The Moshi Urban and Moshi Rural Districts were defined based on the administrative divisions of Tanzania. Briefly, 30 (66.7%) of the 45 wards were selected using a population-weighted random sampling method. In each selected ward a starting point was chosen arbitrarily while touring the ward on foot by a member of the study team who was not previously familiar with the area. A direction was similarly chosen and the first 27 households along that direction from the starting point were included in the survey. Questions relating to health care–seeking behaviour in the event of febrile illness were used to identify participants likely to present to KCMC or MRRH. These questions included, ‘To which facility would you go if you were unwell with a fever lasting ≥3 days?’.^11,18^

### Incidence calculation

**Figure 2.**
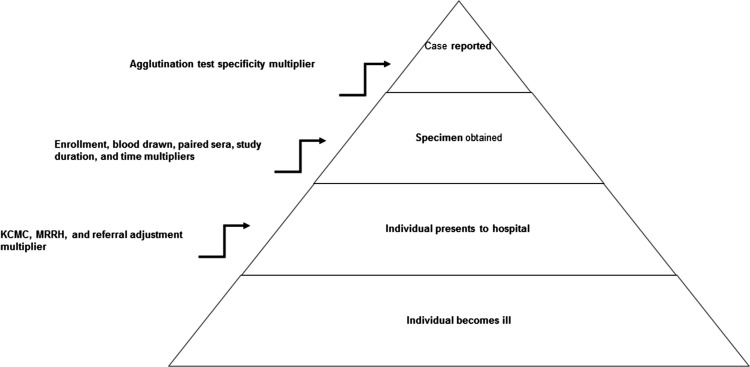
Surveillance pyramid showing multipliers used to account for incomplete case identification. Modified from Biggs HM, Hertz JT, Munishi OM, et al. Estimating leptospirosis incidence using hospital-based surveillance and a population-based health care utilization survey in Tanzania. PLoS Negl Trop Dis 2013;7(12):e258.

Incidence calculation was based on the absolute number of hospital patients meeting the brucellosis case definition and on multipliers derived from the fever surveillance and the health care utilization survey. Multipliers account for brucellosis cases that were potentially missed in the stages of reporting (Figure 2) and are the multiplicative inverse of the relevant proportions.

#### Multipliers

The following multipliers were calculated: (1) KCMC multiplier and MRRH multiplier, to account for health care–seeking preferences and cases potentially missed due to selection of health care facilities not under surveillance. The KCMC multiplier and MRRH multiplier were derived based on head-of-household responses to the question: ‘What will you do if a member of this household has elevated body temperature for ≥3 days?’; (2) referral adjustment multiplier for the period 2007–2008, to adjust for patients transferred to KCMC from another inpatient hospital, given that transfer may not reflect a patient’s preference of health care facility; (3) enrolment multiplier, to account for patients who were eligible but did not enrol in the hospital-based fever surveillance for any reason; (4) blood drawn multiplier, to account for patients for whom the blood volume obtained was insufficient for brucellosis serology; (5) study duration multiplier for the period 2012–2014, to calculate annual incidence from a study that enrolled for 27 months; (6) time multiplier, to account for fever surveillance enrolment 5 of 7 days of the week and (7) agglutination test specificity multiplier, to account for MAT specificity (96.1%).^19^

#### Population denominators

Brucellosis incidence was calculated by age group as follows: 0–4, 5–14 and ≥15 years. As age-specific population data were not available from the 2012 Tanzania National Census, we multiplied age-specific proportions from the 2002 Tanzania National Census by the 2012 Tanzania National Census population total to estimate age-specific populations.^11,20–21^

### Sensitivity analysis

To assess the sensitivity of our incidence estimates, we performed a one-way sensitivity analysis by varying (1) hospital multipliers according to answers to alternative relevant questions in the health care utilization survey that might also reflect the behaviour of participants, (2) diagnostic test multipliers by using a range of alternative sensitivity and specificity values for agglutination tests from the published literature^22–25^ and (3) population denominators for the period 2007–2008 by estimating the Moshi Urban and Moshi Rural populations in the period 2007–2008 as the mean of the 2002 population and the 2012 population in Moshi Urban and Moshi Rural Districts.^11^

### Statistical analysis

Data were entered into an Access database (Microsoft, Redmond, WA, USA) using the Cardiff Teleform system (Cardiff, Vista, CA, USA). Incidence calculations were done using Excel 2016 (Microsoft). Other analyses were performed using SAS Enterprise Guide, version 7.1 (SAS Institute, Cary, NC, USA).

## Results

### Hospital-based fever surveillance

In the period 2007–2008, 1310 patients were eligible for enrolment and 870 (66.4%) participated in the study. Of these participants, 588 (67.6%) were from the Moshi Urban or Moshi Rural Districts. Blood cultures were collected for 585 (99.5%) of these 588 participants. All the participants were inpatients. Of these, 249 (42.6%) were aged 0–4 years, 58 (9.9%) 5-14 years, and 278 (47.5%) ≥15 years. None had a blood culture positive for *Brucella* spp. Among 585 patients residing in the study area and for whom a blood culture was collected, 314 (53.7%) had paired sera tested. Of those with paired sera tested, 13 (4.1%) of 314 met the case definition for brucellosis.

During the period 2012–2014, 2962 patients were eligible for enrolment and 1416 (47.8%) participated in the study. Of these participants, 1115 (78.7%) were from the Moshi Urban or Moshi Rural Districts. Blood cultures were collected for 1095 (98.2%) of 1115 participants. Outpatients accounted for 467 (42.6%) of the participants for whom blood cultures were collected. Of the 1,095 participants, 409 (37.4%) were aged 0-4 years, 110 (10.0%) 5–14 years, and 576 (52.6%) ≥15 years. None had a blood culture positive for *Brucella* spp. Of those residing in the study area and for whom a blood culture was collected, 632 (57.7%) had paired sera tested. Of those with paired sera tested, 32 (5.0%) met the case definition for brucellosis. Of the 32 patients with a diagnosis of brucellosis, 9 (28.1%) were outpatients. Overall, 45 patients had a laboratory-confirmed diagnosis of brucellosis in the study periods. Of these, 41 (91.1%) had a syndrome clinically compatible with brucellosis, as defined by the US CDC. Among the 45 brucellosis cases identified in the two study periods, the median duration of illness before presentation was 5 days (interquartile range [IQR] 4–7) in the period 2007–2008 and 7 days (IQR 3–14) in the period 2012–2014. For all 45 brucellosis cases identified from both the study periods, brucellosis was never recorded as a clinical diagnosis by the treating physician, neither at admission or at discharge. The most common clinical diagnoses were as follows: 20 (44.4%) malaria, 6 (13.3%) pneumonia, 4 (8.9%) septicaemia, 4 (8.9%) gastroenteritis, 3 (6.7%) upper respiratory tract infection and 3 (6.7%) urinary tract infection. The five remaining cases were given the following clinical diagnoses by the treating physician: anaemia, diabetes, meningitis, paratyphoid fever and pruritus. In both study periods, none of the patients with a laboratory diagnosis of brucellosis received one of the recommended first-line combination antibacterial regimens (doxycycline plus aminoglycoside or doxycycline plus rifampin); five patients with a laboratory diagnosis of brucellosis (one in the period 2007–2008 and four in the period 2012–2014) were treated with ciprofloxacin monotherapy, but the duration of the treatment is unknown. Of the patients who did not have a laboratory diagnosis of brucellosis, only one was treated with a recommended first-line antimicrobial regimen for brucellosis (doxycycline plus gentamicin).

### Health care utilization survey

A total of 810 households were sampled, comprising 3919 household members. All households had at least one member ≥15 years of age, 361 (44.6%) had at least one member between 5 and 14 years of age and 198 (24.4%) had at least one member <5 years of age. Table 1 illustrates the responses to the question ‘What will you do if a household member has a fever for ≥3 days?’.

**Table 1.** Responses to the question in the health care utilization survey, ‘What will you do if a household member has a fever for ≥3 days?’

Age (years)	Household members, n	Household members going to KCMC, n (%)	Household members going to MRRH, n (%)	Household members not going to KCMC or MRRH, n (%)
<5	198	17 (8.6)	67 (33.8)	114 (57.6)
5–14	361	10 (2.8)	137 (38.0)	214 (59.3)
≥15	810	35 (4.3)	299 (36.9)	476 (58.8)

### Incidence calculation and sensitivity analysis

Multipliers were calculated (Table 2). By applying multipliers to confirmed cases, we estimated an overall incidence of brucellosis of 35 cases per 100 000 persons annually in the period 2007–2008 (Table 3) and 33 cases per 100 000 persons annually in the period 2012–2014 (Table 4). Brucellosis incidence appeared to be higher in the Moshi Urban District than the Moshi Rural District in the period 2007–2008, at 53 cases per 100 000 persons annually compared with 25 cases per 100 000 persons annually, respectively, and in the period 2012–2014 at 88 cases per 100 000 persons annually compared with 14 cases per 100 000 persons annually, respectively. The variation of hospital multipliers, diagnostic test multipliers and population denominators in the period 2007–2008 resulted in an annual brucellosis incidence ranging from 32 to 93 cases per 100 000 population. The variation of hospital multipliers and diagnostic test multipliers in the period 2012–2014 resulted in an annual brucellosis incidence ranging from 30 to 89 cases per 100 000 population. In both study periods the highest incidence estimates were obtained when hospital multipliers were derived from the question ‘What will you do if a household member has a fever?’. In contrast, the lowest brucellosis incidence estimates were associated with the use of the lowest plausible MAT specificity estimate (Table 5).

**Table 2.** Derivation of multipliers to estimate the incidence of brucellosis in the Moshi Rural and Moshi Urban Districts, Kilimanjaro Region, Tanzania

	Multiplier equation	Multiplier for period			
		2007–2008		2012–2014	
KCMC multiplier	=no. of households interviewed/no. of households seeking care at KCMC for fever ≥3 days				
age <5 years		198/17	11.63	198/17	11.63
age 5–14 years		361/10	36.10	361/10	36.10
age ≥15 years		810/35	23.10	810/35	23.10
MRRH multiplier	=no. of households interviewed/no. of households seeking care at MRRH for fever ≥3 days				
age <5 years		198/67	3.00	198/67	3.00
age 5–14 years		361/137	2.64	361/137	2.64
age ≥15 years		810/299	2.71	810/299	2.71
Referral adjustment multiplier	=no. of study patients admitted to KCMC without referral from another facility/no. of patients admitted to KCMC				
age <5 years		166/249	0.67	N/A	N/A
age 5–14 years		40/55	0.80	N/A	N/A
age ≥15 years		72/94	0.77	N/A	N/A
Enrolment multiplier	=no. of eligible patients/no. of patients enrolled in fever surveillance	1310/870	1.51	2394/1420	1.69
Blood drawn multiplier	=no. of patients included in the incidence study/no. of patients for whom serology was performed				
age <5 years		249/232	1.07	409/335	1.22
age 5–14 years		58/57	1.02	110/108	1.02
age ≥15 years		278/274	1.01	576/554	1.04
Study duration multiplier	=no. of months per year/study duration (in months)	N/A^a^	N/A	12/27	0.44
Time multiplier	=no. of days in a week/no. of enrolment days per week	7/5	1.40	7/5	1.40
Paired sera multiplier	=no. of patients included in the incidence study for whom serum was collected/no. of patients included in the incidence study with paired sera	563/314	1.79	997/632	1.58
AT specificity multiplier	=specificity	0.96	0.96	0.96	0.96

AT: agglutination test; N/A: not applicable.

^a^Study duration multiplier not applicable for 2007–2008 study period because the study enrolment lasted 1 year and 15 days, therefore it was not necessary to annualize the case numbers.

**Table 3.** Annual incidence of brucellosis in the Moshi Rural and Moshi Urban Districts, Kilimanjaro Region, Tanzania, 2007–2008, based on the question, ‘To which facility would you go if you were unwell with a fever lasting ≥3 days?’

Age group (year)	KCMC crude cases	KCMC adjusted cases	MRRH crude cases	MRRH adjusted cases	Estimated annual cases	Population	Annual incidence per 100 000
<5	4	30	N/A	N/A	125	82 016	153
5–14	N/A	N/A	N/A	N/A	0	179 387	0
≥15	2	34	7	19	105	389 625	27
Overall					230	651 028	35

KCMC: Kilimanjaro Christian Medical Centre; MRRH: Mawenzi Regional Referral Hospital; N/A: not applicable.

**Table 4.** Annual incidence of brucellosis in the Moshi Rural and Moshi Urban Districts, Kilimanjaro Region, Tanzania, 2012–14, based on the question, ‘To which facility would you go if you were unwell with a fever lasting ≥3 days?’

Age group (year)	KCMC crude cases	KCMC adjusted cases	MRRH crude cases	MRRH adjusted cases	Estimated annual cases	Population	Annual incidence per 100 000
<5	N/A	N/A	3	11	22	82 016	27
5–14	N/A	N/A	2	10	17	179 387	9
≥15	6	133	21	72	177	389 625	45
Overall					216	651 028	33

N/A: not applicable.

**Table 5.** Sensitivity analysis to assess the precision of brucellosis incidence estimates in the Moshi Urban and Moshi Rural Districts, Kilimanjaro Region, Tanzania, 2007–2008 and 2012–2014, based on the variation of hospital multipliers, diagnostic test multipliers and population denominators

	Estimated incidence of brucellosis per 100 000 population per year	
	Period 2007–2008	Period 2012–2014
Variation in hospital multipliers based on varying the question from the health care utilization survey^a^		
Question: What will you do if a household member has a fever lasting <3 days?	66	56
Question: What will you do if a household member has a fever (duration not specified)?	93	89
Variations in estimation of specificity of agglutination test		
Lowest plausible estimate	32	30
Single titre ≥1:160: 88%		
Highest plausible estimate	37	35
Single titre ≥1:160: 100%		
Variations in census data		
Total population for the period 2007–2008 is the mean of the census population in 2002 and in 2012 (=598 098)	38	N/A

N/A: not applicable.

^a^Hospital multipliers are derived from questions included in the health care utilization survey (see Methods) and account for cases potentially not captured by our surveillance sites (KCMC and MRRH).

## Discussion

We found a moderate incidence of brucellosis in the Kilimanjaro Region of Tanzania of 35 cases per 100 000 in the period 2007–2008 and 33 cases per 100 000 in the period 2012–2014. These estimates suggest that brucellosis is stably endemic in the study area and provide important epidemiologic information on the human health impact of this disease in the Kilimanjaro Region.^26^

Our study is among the first to estimate brucellosis incidence in a sub-Saharan African country based on health care facility–based surveillance and health care utilization data.^26^ While there are a number of studies of brucellosis prevalence, as far as we know the only available incidence estimates of brucellosis in sub-Saharan Africa come from Chad, where an incidence of 35 cases per 100 000 person-years was derived from a seroprevalence of 3.8%, assuming a fixed proportion of clinical cases among seropositives (10.0%) and a fixed duration of seropositivity.^5^ We suggest that our study based on detection of cases over many years of surveillance provides a more robust means of estimating incidence and we hope that similar studies will be undertaken elsewhere in sub-Saharan Africa. Furthermore, the sensitivity analysis showed that when the uncertainty in key parameters was considered, the incidence estimates did not vary substantially. Our estimate was most sensitive to varying the health care utilization survey questions about fever. The higher incidence estimates derived by investigating health care–seeking behaviour for fever of unspecified duration and for fever lasting <3 days reflects the fact that patients are less likely to present to tertiary hospitals in cases of shorter durations of fever. We think that the estimates based on fever of unspecified duration and on fever lasting <3 days are less accurate than our final estimate, as 85% of the patients diagnosed with brucellosis reported a fever of at least 3 days.^11^ These durations are compatible with data available in the literature.^27^

Our study raises several other interesting points. First, our estimates of brucellosis incidence varied substantially among age groups. While these variations may be related to inadequate power or study design, the apparent low incidence of brucellosis among those 5–14 years of age may be due to differences in exposure or host factors by age. Second, we estimated an apparently higher incidence of brucellosis in the Moshi Urban District compared with the Moshi Rural District. It is important to note that this estimate is based on the assumption that the age distribution was similar in the Moshi Urban and Moshi Rural Districts during the two study periods. If true, this finding suggests that urban residents may be at higher risk of exposure to *Brucella* spp. than rural persons in this area, perhaps pointing to exposure pathways that involve wide food distribution networks. Third, brucellosis was never recorded as a clinical diagnosis by the treating physician, neither at admission nor at discharge. This is likely due to a low index of suspicion for brucellosis among treating physicians, compounded by the lack of accurate diagnostic tests for brucellosis. Finally, our study highlights that employing rigorous serologic diagnostics is important for epidemiologic research on brucellosis. While culture of blood, bone marrow and other normally sterile sites provides the most specific diagnosis, the limited sensitivity of blood culture for *Brucella* spp. detection is well-documented.^28^ Relying solely on blood culture for surveillance of brucellosis may lead to substantial underestimates of incidence.

Our estimates represent our best effort to evaluate the incidence of human brucellosis in sub-Saharan Africa, but we recognize a number of limitations. First, our case definition of brucellosis was based only on laboratory parameters, since comprehensive clinical information were lacking for the study period 2007–2008. Despite the fact that not all variables included in the CDC case definition of brucellosis were assessed during the study period 2007–2008, 41 (91.1%) of the 45 laboratory-confirmed brucellosis cases had a syndrome clinically compatible with brucellosis as defined by the CDC. While incidence is fundamental to understanding the burden of brucellosis, we did not evaluate other components of disease burden, such as attributed disability and case:fatality ratio. Specifically, the assessment of disability was beyond the scope of this research, and case fatality ratios were not estimated because no patients with a blood culture positive for *Brucella* spp. went on to die of their illness, and serologic case confirmation requires survival for collection of serum 4–6 weeks after enrolment. The multiplier method employed in this study has been described and applied to estimate the incidence of febrile illnesses in settings where resources and infrastructure for active population-based surveillance are insufficient.^8–10,29^ However, several assumptions were made in the derivation and use of multipliers. In particular, we assume that patients presenting at the study sites were representative of the household members sampled in the health care utilization survey and that the care-seeking behaviour of those surveyed was representative of the overall population.^11^ Finally, the assessment of risk factors for human brucellosis (e.g., occupational exposures, foodborne exposures, livestock husbandry practices, etc.) as well as evaluation of the prevalence of brucellosis among animals in the study area were beyond the scope of this project.^30^

## Conclusions

Our study highlights that brucellosis is endemic in the Kilimanjaro Region of Tanzania. Efforts to identify the reservoirs, sources and modes of transmission are needed to inform control strategies. Furthermore, our study reveals the importance of developing clinical risk stratification algorithms and brucellosis diagnostic capacity to inform the treatment of febrile illness in Tanzania and elsewhere in sub-Saharan Africa.
